# Therapeutic Application of Blisters

**Published:** 1900-03

**Authors:** C. D. Hurt

**Affiliations:** Atlanta, Ga.


### THERAPEUTIC APPLICATION OF BLISTERS.

By C. D. HURT, M.D.,

Atlanta, Ga.

Much has been said pro and con on the subject of blisters as therapeutical agents. They have been lauded by some and decried by others. They are of incalculable value in the judgments and experiences of some practitioners, and are regarded as cruel and criminal by others. Fairness in judgment rendered in each case depends largely upon the environments and prejudices accompanying man's experience in life. Every claim of protestation against the use of blisters has in it a part of truth, which entitles it to credence and consideration. Every imagination which enters into the minds of different practitioners is not based either upon rational theory or sound experience. The diversities of opinions are in a large measure the product of imperfect investigation, which has gone on in the minds of men for a considerable length of time. The pendulum of prejudice has swung too far away from the plumb line, because of the indiscreet and unexplained positions of men who have been brought into prominence in the medical profession through the merits of accomplishment, well deserved in certain lines. It has occurred many times that men who have occupied the position of teachers have allowed themselves to be guilty of the fault of making a protest to the therapeutic use of an agent in a specific disease, and leaving the impression upon the minds of the students that this protest is applied to the treatment of all diseases. I note a quotation in the *Southern Medical Record* of January, 1899, from Dr. Bull, in which he expresses himself, if correctly quoted, in the following sentences:

"Blisters often produce open wounds, which facilitate secondary infection or the absorption of cantharides. 2. They tend to cause inflammation of the kidneys and bladder, and have a general congestive action. 3. They should be discarded from treatment in pneumonia and pleurisy, because, though they increase pulmonary ventilation, they increase also pulmonary congestion. 4. Blisters tend to arrest excretion by the kidneys, so important in all infectious diseases, and this is epecially harmful in those naturally causing albuminura. Instead of aiding the excretion of toxines, blisters are likely to produce a fresh inoculation. 5. The only real use of blisters is in their revulsive and analgesic action."

Now, if Dr. Bull is correctly quoted, he forgets that blisters properly produced and treated, rarely leave an open wound, and on this account rarely produce a condition in which infection may take place. There was a time in the experience of the profession when opened wounds from cantharidal vesication was the rule and not the exception. This rule was the result of both intentional and unintentional acts of the physician. Intentional when he desired to make an issue from a prolonged and irritating sore, and when he desired to medicate the patient by direct application of the medicine to this open wound. The unintentional was when our skill in removal of the cantharidal plasters did not prevent our carrying with it the raised epidermis. Having other and better means of applying medication, and finding now that we discard the necessity for open blisters, we no longer intentionally convert them into open wounds. In the advancement of medical and pharmaceutical skill ignorance is no longer an excuse for converting blisters into open wounds. Dr. Bull also objects to the disturbance of the kidneys as secretive organs and of the bladder as an excretive organ. It is true that the constitutional effect of cantharides causes congestion of the kidney and spasmodic irritation of the bladder and urethra. But we must bear in mind that the absorption of cantharides from a plaster is limited very much by the local action in cauterizing the epidermis and causing an exudate. It is the exception and not the rule that a sufficient amount of cantharides is absorbed to produce objectionable constitutional effect.

Dr. Bull further states that blisters cause a general congestive action. Just what the doctor means by this statement is hard to understand. Surely he does not mean that a blister applied to the knee will produce congestion of the lungs, brain or heart. The term general congestion, if applied to the circulation, is an error. Any effect must be the result of disturbance of the nervous system. Dr. Bull testifies that pneumonia and pleurisy should be treated without the use of blisters, because they do increase pulmonary ventilation and at the same time increase pulmonary congestion. To my mind this statement is paradoxical and wholly inconsistent. To increase ventilation means to increase receptive capacity by the removal of something already occupying the space to be ventilated, whether this occupant be air, water, blood or solids. How can ventilation be increased if there is increased congestion—influx of a greater amount of blood into the pulmonary tissues? Again, the pulmonary depletion caused by the flow of serum on the blistered surface lessens the congestion. There is no continued pain from a well-managed blister, and hence it cannot be said, *ubi irritatio, ibi fluxus.*

The prejudices which existed against venesection were the result of the indiscriminate use and almost universal application without regard to dosage or disease, the effect upon the consititution, and so on. No argument is needed to convince us of the potency of any active agent to do evil as well as good. No argument is needed further than the experiences which we already have to convince us of the danger arising from the excessive use of many therapeutical agents which are benign in their effect when given in medicinal quantities. The assurances of good through this medium of venesection have all been lost sight of because of the strong argument of universal prejudice. Now that the smoke has cleared away and the minds of the public have been brought to see the truth in the careful practice of this agency, the medical world is tending to accord it a sufficient place under scientific application. It will be none the less true as time shall remove both ignorance and prejudice in regard to the use of blisters. Between empiricism and a thorough knowledge of the physiological action of drugs, there is a point where clouds gather and where the incredulous or unfortunate raise their arms in full defiance of whatever may be said pro or con.

The prejudice against cantharidal vesication grows out of several causes: First, a lack of a thorough knowledge of the pathology and temperament of the patient; second, the heretofore universal application from empiricism instead of properly selected cases from a scientific standpoint; third, a timidity which prevents the timely application of a sufficiently extensive blister to accomplish satisfactory results; fourth, the error of converting a vesicated surface into an open suppurating wound, creating pain and shock to the nervous system; fifth, allowing the cantharides to remain too long, burning deep into the tissues; sixth, the modern teaching protesting against their use under any circumstances.

Some methods of preparing and applying cantharides I find very objectionable and disappointing. Cantharidal ointment, in which the cantharides is incorporated with yellow wax and olive oil, is not satisfactory, because it liquefies or softens too much with body heat and smears on the skin at points away from the plaster; and the olive oil, being a solvent of cantharides, is liable to concentrate the strength of the drug at a dependent portion of the body. Thus you are liable to have anything but a uniform blister. The proprietary plaster, or that made by the manufacturers and kept in stock, is liable to, and does, deteriorate. I have kept such in contact with the skin twenty-four hours with no result. The cantharidal collodion is more reliable, and on certain portions of the body of irregular surface, is a satisfactory way of applying cantharides.

The cerate of the extract of cantharides of the U. S. P., while a little more trouble to the pharmacist to prepare and requiring to be spread when needed for application, is much more reliable, acts in a shorter time, and, if spread with uniform thickness, makes a blister of uniform depth, and excludes the air so that the patient suffers no pain until removed, and then only momentary if a warm poultice is promptly applied. A piece of thin muslin should be laid over the surface of the cerate before applying to insure complete and easy removal.

*Atlanta, Ga., February 15, 1900.*

